# Non-pathogenic tissue-resident CD8^+^ T cells uniquely accumulate in the brains of lupus-prone mice

**DOI:** 10.1038/srep40838

**Published:** 2017-01-18

**Authors:** Peter A. Morawski, Chen-Feng Qi, Silvia Bolland

**Affiliations:** 1 Laboratory of Immunogenetics, Division of Intramural Research, National Institute of Allergy and Infectious Diseases, NIH, Rockville, MD 20852, USA

## Abstract

Severe lupus often includes psychiatric and neurological sequelae, although the cellular contributors to CNS disease remain poorly defined. Using intravascular staining to discriminate tissue-localized from blood-borne cells, we find substantial accumulation of CD8^+^ T cells relative to other lymphocytes in brain tissue, which correlates with lupus disease and limited neuropathology. This is in contrast to all other affected organs, where infiltrating CD4^+^ cells are predominant. Brain-infiltrating CD8^+^ T cells represent an activated subset of those found in the periphery, having a resident-memory phenotype (CD69^+^CD122^−^PD1^+^CD44^+^CD62L^−^) and expressing adhesion molecules (VLA-4^+^LFA-1^+^) complementary to activated brain endothelium. Remarkably, infiltrating CD8^+^ T cells do not cause tissue damage in lupus-prone mice, as genetic ablation of these cells via β2 m deficiency does not reverse neuropathology, but exacerbates disease both in the brain and globally despite decreased serum IgG levels. Thus, lupus-associated inflammation disrupts the blood-brain barrier in a discriminating way biased in favor of non-pathogenic CD8^+^ T cells relative to other infiltrating leukocytes, perhaps preventing further tissue damage in such a sensitive organ.

The pathogenesis of systemic lupus erythematosus (SLE) is thought to involve a combination of genetic and environmental factors that produces a myriad of symptoms depending on the various organs affected^1^,^2^. Neuropsychiatric lupus is one prevalent manifestation of the disease in humans, including symptoms such as headaches, cognitive dysfunction, or affective disorders^3^, some of which have been recapitulated in a mouse model of lupus^4^. In addition, cerebral vasculitis, the swelling of the endothelial cells lining blood vessels of the brain, has been associated with development of more severe lupus^5^,^6^. As is the case with many aspects of SLE, autoantibodies have been proposed to play a dominant role in the development of brain pathology^7^,^8^,^9^,^10^. However, there remains little known about the cellular contributors to lupus disease activity in CNS tissue, particularly concerning the role of T lymphocytes. Previous studies that identified tissue-infiltrating lymphocytes in lupus-prone animals relied largely on histological analysis, which is largely qualitative and allows only limited phenotyping, while analysis performed following transcardial perfusion is known now to leave many cells behind in the vasculature complicating the interpretation of flow cytometry data^11^. We bypass this issue using a recently described technique in which a simple intravascular stain with a fluorescently-labeled antibody marking hematopoetic cells of interest allows discrimination by flow cytometry between tissue-resident and blood-borne cells^11^.

The blood-brain barrier (BBB) and the blood-cerebrospinal fluid barrier (BCSFB) regulate the diffusion of cells and water-soluble molecules into the central nervous system (CNS). These structures are composed of a single layer of endothelial cells linked by a complex network of tight junctions. While the BBB and BCSFB are largely not permissive, T cells can enter and survey the brain in the absence of neuroinflammation and barrier damage. However, these T cells show little motility and exit quickly unless they encounter a cognate antigen^12^,^13^. Typically, lymphocytes entering the CNS do so in response to inflammation resulting from infection^14^ or due to autoimmune pathology^15^. However, most studies on brain-infiltrating T cells in mouse models of CNS-based autoimmunity have focused on the role of antigen-specific CD4^+^ T cells in diseases such as multiple sclerosis (MS).

CD8^+^ T cells have been implicated in aspects of several CNS-based autoimmune disorders, for example in neural lesion formation during MS^16^. They also have been shown to accumulate in the brain in a mouse model of amyotrophic lateral sclerosis (ALS)^17^. Equal ratios of CD4^+^ and CD8^+^ T cells have been described in the choroid plexus of MRL/lpr lupus-model mice, though the phenotype and function of these cells were not defined^18^. The most compelling data implicating CD8^+^ T cells in autoimmune disease comes from a transcriptome analysis of human peripheral blood from SLE and anti-neutrophil cytoplasmic antibody (ANCA)-associated vasculitis (AAV) patients^19^. This study highlighted a disease-associated CD8^+^ memory T cell signature that included elevated expression of IL-7 receptor signaling molecules. Another example of the importance of memory CD8^+^ lymphocytes in CNS inflammatory and autoimmune disease comes from the characterization of tissue-resident memory cells (Trm), though direct evidence of Trm in lupus has not been reported^20^. Overall, the contribution of CD8^+^ T cells to tissue-specific immune responses during lupus, whether they are deleterious or protective, particularly in the brain, is understudied. Here, we use recently introduced techniques to identify and characterize immune cell populations infiltrating brain tissue in the context of systemic lupus disease. Our results indicate a differential ability for lymphocytes to infiltrate the CNS in a lupus environment. This selectivity might explain the limited pathology in the brain relative to other organs.

## Results

### Tissue-resident memory CD8^+^ T lymphocytes accumulate in the brain parenchyma of lupus-prone mice

**Figure 1:**
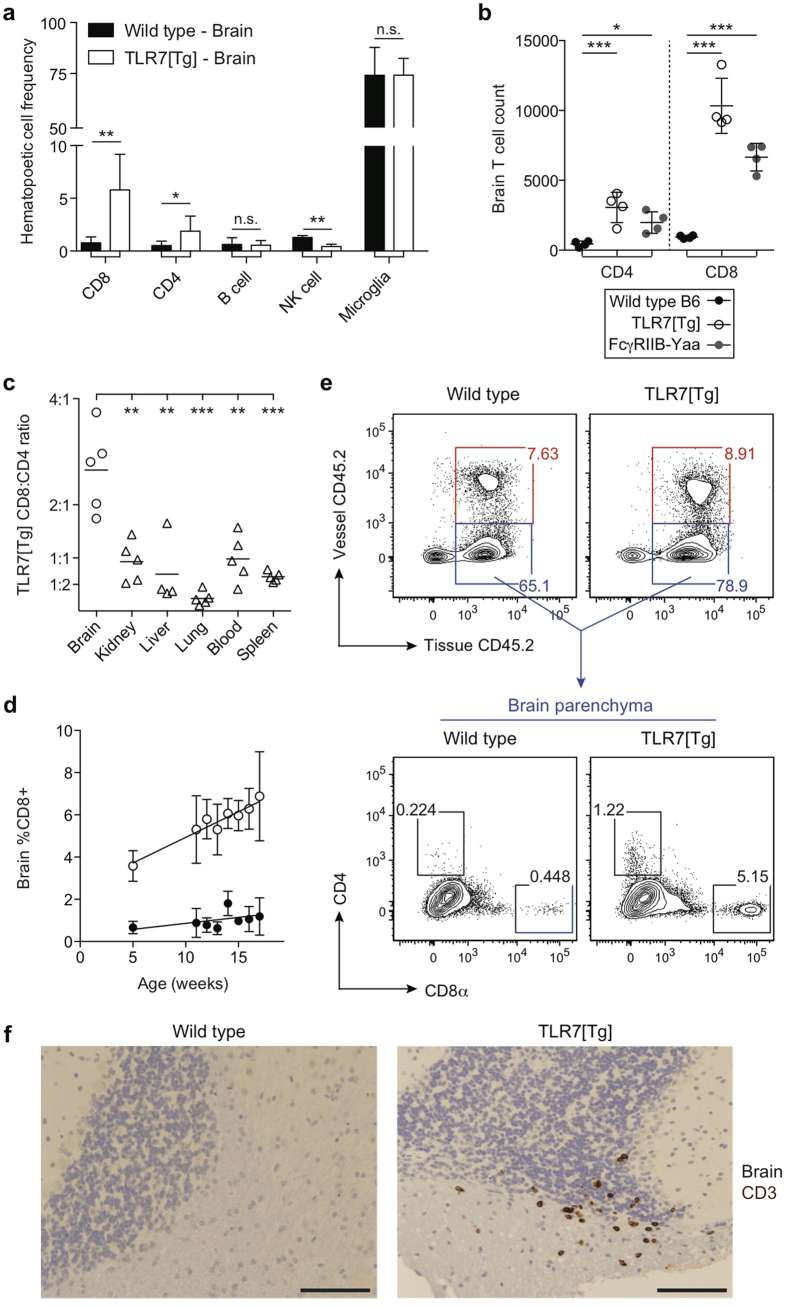
The brains of lupus mice uniquely contain a population of CD8^+^ T cells that accumulates with age. (**a–e**) Flow cytometric analysis of hematopoietic cells purified from the brain and gated on live, singlet, CD45^+^. (**a**) TLR7[Tg] and WT mouse brain samples are tested for infiltration of T cells (CD8^+^ or CD4^+^), B cells (B220^+^, CD19^+^), NK cells (NK1.1^+^, NKp46^+^), and microglia (CD45^lo^, CD11b^+^). Data are representative of at least two experiments, and at least three mice per cell type shown. (**b**) Absolute count of live CD8^+^ and CD4^+^ T cells in the brains of TLR7[Tg], R2-Yaa, and WT mice. Data are representative of at least three experiments. (**c**) CD8^+^/CD4^+^ T cell ratio in indicated organs following fluorescent antibody labeling of blood and tissue of TLR7[Tg] and WT mice. Data are representative of at least three independent experiments. (**d**) Live CD8^+^ T cell quantification in the brain of TLR7[Tg] and WT mice. Data are pooled from >10 experiments performed using TLR7[Tg] mice. Each time point includes data from at least three mice. (**e**) Discrimination of T lymphocytes associated with the brain blood vessels or embedded in the parenchyma of TLR7[Tg] and WT animals following *in vivo* antibody vessel labeling. Data are representative of three independent experiments. (**f**) Immunohistochemical staining of brain tissue for hematoxylin and CD3 from 4% formaldehyde perfused TLR7[Tg] and WT mice shows presence of lymphocytes in brain parenchyma. Representative image of T cell infiltration of the cerebellum includes hematoxylin staining of ependymal cells. Scale bars, 50 μm. Error bars indicate mean + s.d. *P ≤ 0.05, **P ≤ 0.01, and ***P ≤ 0.001 (Student’s t test).

To determine whether there is any infiltration of lymphocytes into the brain during lupus disease, mononuclear cells were purified from the brains of C57BL/6 wild-type (WT) and TLR7 transgenic (TLR7[Tg]) mice expressing 8–16 copies of the Tlr7 gene^21^. Flow cytometry analysis revealed a profound increase in brain-infiltrating CD8^+^ and CD4^+^ T cells relative to other hematopoietic populations in TLR7[Tg] mice, while microglia, the resident macrophages of the brain, were unchanged compared to WT controls (Fig. 1a). The percentage and absolute number of CD8^+^ T cells exceeded that of CD4^+^ T cells in the brain of TLR7[Tg] mice by an average of 3-fold (Fig. 1a,b). Strikingly, the bias of infiltrating CD8^+^ T cells versus CD4^+^ T cells was only found in the brain and not in any other organ we examined in TLR7[Tg] mice (Fig. 1c). This CD8^+^ to CD4^+^ T cell bias exists throughout the brain, in the olfactory bulbs, cortex, cerebellum, and mid-brain (Supplementary Fig. 1a), and the CD8^+^ T cells accumulate proportionally relative to the size of each part of the brain measured (Supplementary Fig. 1b–d). Brain-infiltrating CD8^+^ T cells in TLR7[Tg] mice were present as early as five weeks of age and accumulated as the mice aged (Fig. 1d). We also found an increase in CD8^+^ and CD4^+^ T cells in the brains of another lupus model, FcγRIIB[KO]-Yaa (R2-Yaa) (Fig. 1b)^22^. Using *in vivo* vessel-labeling with fluorescent antibodies^11^,^23^ we distinguished lymphocytes in the brain tissue of TLR7[Tg] mice from those adhering to the blood vessel endothelium, demonstrating the lymphocyte accumulation occurs within the brain parenchyma (Fig. 1e). These data are supported by immunohistochemical staining for T cells on brain and spinal cord sections isolated from both TLR7[Tg] and WT mice, which demonstrates the presence of CD3^+^ cells embedded in cortex, cerebellum, choroid plexus, and spinal cord (Fig. 1f; Supplementary Fig. 1e).

We next wanted to test what factors affected the size of the brain-CD8^+^ T cell pool. We found that the accumulation of CD8^+^ T cells in the brain of TLR7[Tg] mice was not the result of either increased proliferation as measured by BrdU (Supplementary Fig. 2a,b) or altered apoptosis as measured by *ex vivo* expression of caspase and annexin V (Supplementary Fig. 2c,d) relative to peripheral CD8^+^ T cells from TLR7[Tg] and WT controls. We also did not observe any correlation between the weight of the brain and number of CD8^+^ T cells present in the organ (Supplementary Fig. 2e). In addition, though aspects of the disease in TLR7[Tg] mice include both splenomegaly and lymphoproliferation^21^, there was no correlation between the size of the spleen and the total mononuclear cell count in the brain (Supplementary Fig. 2f). Importantly, absolute CD8^+^ T cell numbers remain largely unchanged in the spleen and blood of both TLR7[Tg] and R2-Yaa mice as compared to WT controls^21^, indicating that the bias of infiltrating CD8^+^ T cells in these mice is not simply the result of uncontrolled peripheral lymphocyte expansion.

**Figure 2:**
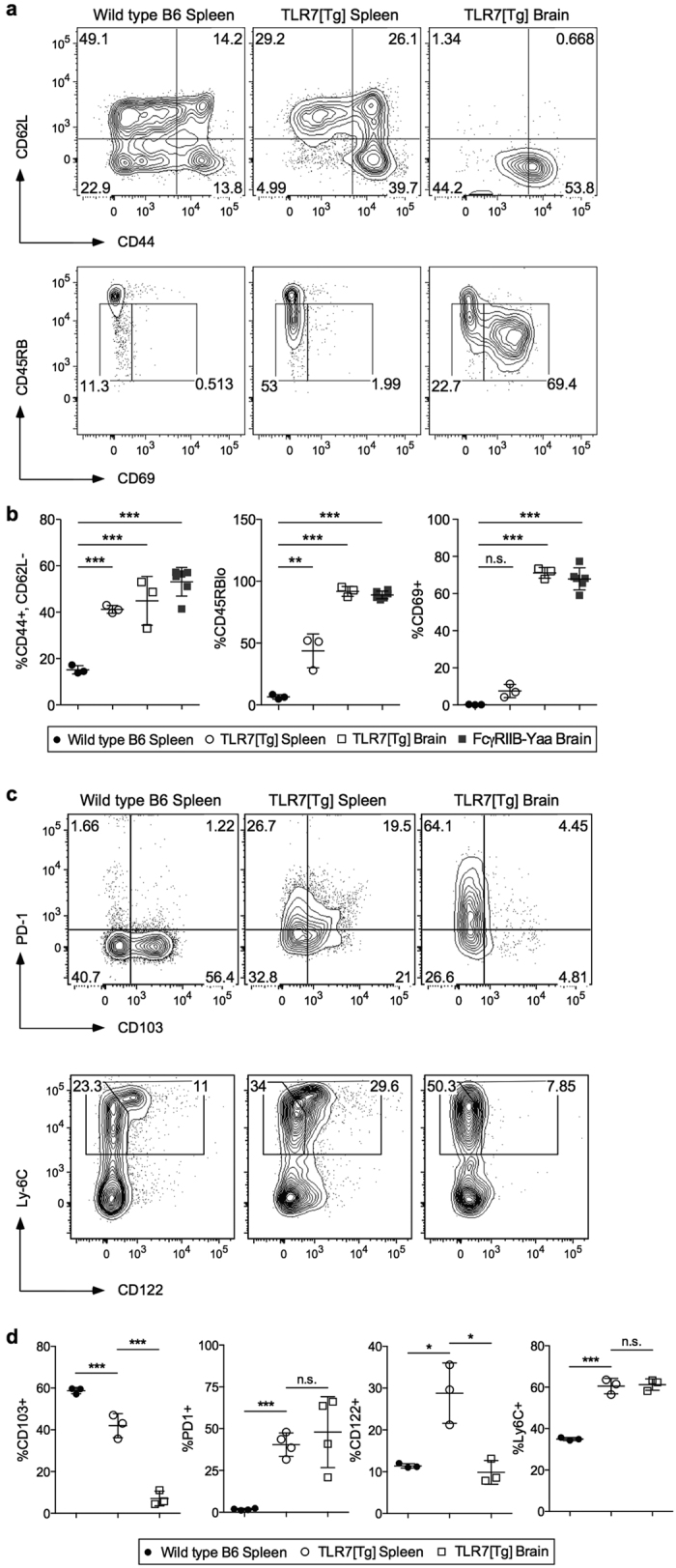
CD8^+^ T cells in the brain have the phenotype of tissue-resident memory cells. Flow cytometric analysis of activation and tissue-resident memory markers on CD8^+^ T lymphocytes in brain and spleen of vessel-labeled or trans-cardially perfused TLR7[Tg], R2-Yaa, and WT mice. All cells gated on CD45^+^, live singlets. (**a,b**) Activated cells are defined by differences in CD8^+^ T cell expression of CD44, CD62L, CD69, and CD45RB. (**c,d**) Tissue-resident memory cells are defined by differences in CD8^+^ T cell expression of PD-1, CD122, Ly-6C, and CD103, as well as CD69 (**a,b**). Data are representative of at least three experiments. Error bars indicate mean + s.d. *P ≤ 0.05, **P ≤ 0.01, and ***P ≤ 0.001 (Student’s t test).

We characterized the CD8^+^ T cells accumulating in the brain of lupus-prone mice by their expression of surface markers previously described for various activated effector T cell subsets, and compared them to CD8^+^ T cells present in the spleen of WT and lupus-prone mice. No comparison of WT brain CD8^+^ T cells was possible due to small cell numbers. Consistent with our previous findings, *ex vivo* splenic CD8^+^ T cells from TLR7[Tg] and R2-Yaa mice have an effector phenotype relative to WT controls as defined by elevated expression of CD44 and a reduction in the expression of both L-selectin (CD62L) and CD45RB (Fig. 2a,b). The population of CD8^+^ T cells in the brains of TLR7[Tg] mice was enriched in expression of these surface proteins, and included greatly elevated expression of CD69 (Fig. 2a,b).

Based on the marked upregulation of CD69, we posited that these CD8^+^ T cells are akin to Trm cells required for alerting and activating the local tissue environment in response to inflammation^24^. Trm cells are defined by upregulation of CD69, PD-1, and in some cases αEβ7 integrin (CD103), as well as downregulation of Ly6C and CD122 expression. The CD8^+^ T cells from the brains of lupus-prone mice exhibited substantial CD69 (Fig. 2a,b) and PD-1 upregulation (Fig. 2c,d) and CD122 downregulation (Fig. 2c,d) relative to peripheral CD8^+^ T cells from the same mice, indicative of a Trm cell phenotype. These cells are largely CD103 negative (Fig. 2c,d), which is similar to Trm cells described previously in barrier tissues of both humans^25^ and mice^26^. Collectively, these data demonstrate that a tissue-resident memory population of CD8^+^ T cells is localized to the brain of lupus-prone mice.

### CD8^+^ T cells from TLR7[Tg] mice home to the brain and have elevated expression of cell adhesion molecules

**Figure 3:**
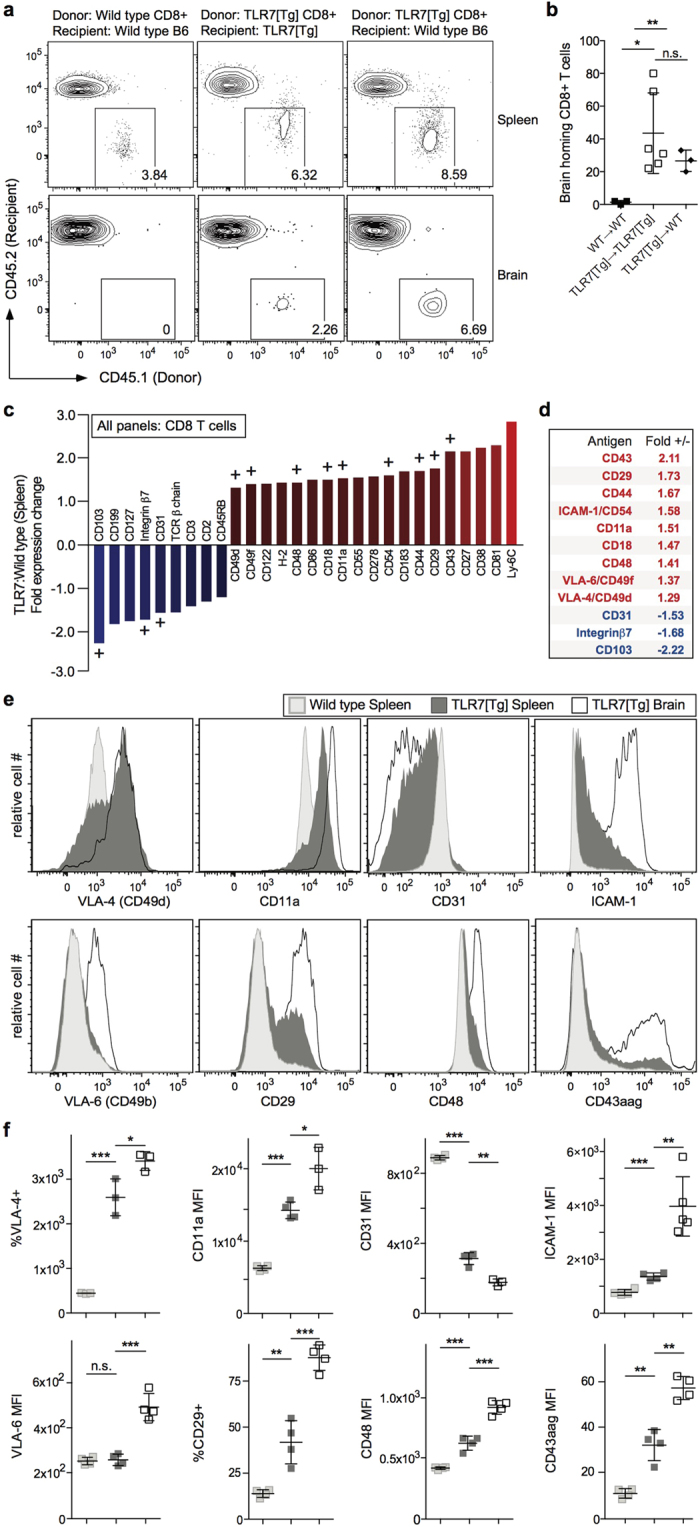
Peripheral CD8^+^ T cells are primed for tissue entry and home to the brain. (**a,b**) Flow cytometric analysis of brain homing CD8^+^ T lymphocytes from vessel labeled or trans-cardially perfused TLR[Tg] and WT mice. Indicated recipient mice were injected with purified peripheral TLR7[Tg] or WT CD8^+^ T cells from congenic mice (CD45.1) as indicated. Data in (**a**) are gated on total CD8^+^ T cells and are representative of two experiments. Graph in (**b**) shows two pooled experiments. Cells gated on live singlet CD8a^+^. (**c,d**) Flow cytometric analysis of comprehensive surface antigen screen performed on TLR7[Tg] and WT mice. Fold change of mean fluorescence intensity for PE-labeled antigens shown. Proteins involved in lymphocyte trafficking and adhesion to tissue endothelium indicated by “+” above fold expression bar. Red, upregulated. Blue, downregulated. (**e,f**) Flow cytometric analysis of indicated trafficking and adhesion molecules significantly upregulated on TLR7[Tg] brain-resident CD8^+^ T lymphocytes compared to peripheral cells TLR7[Tg] and WT mice. Data are representative of at least three experiments. Cells gated on live singlet CD45^+^. Error bars indicate mean + s.d. *P ≤ 0.05, **P ≤ 0.01, and ***P ≤ 0.001 (Student’s t test).

We tested the ability of peripheral CD8^+^ T cells from lupus-prone mice to home to the brain by transferring five million purified CD45.1^+^ splenic TLR7[Tg] CD8^+^ T cells into CD45.2^+^ WT or TLR7[Tg] recipient mice. WT CD45.1^+^CD8^+^ T cells were injected intravenously into WT CD45.2 mice as a control transfer. Six days after the transfer, we detected donor-derived TLR7[Tg] but not wild type CD8^+^ T cells in the brains of both WT and TLR7[Tg] recipients (Fig. 3a,b). Importantly, no neuropathology could be found associated with transferred TLR7[Tg] CD8^+^ T cells (Supplementary Fig. 3a). These data indicate that peripheral non-pathogenic TLR7[Tg] CD8^+^ T cells are more likely to migrate to and enter WT and TLR7[Tg] brains than are WT CD8^+^ cells. To uncover differences between TLR7[Tg] and WT splenic CD8^+^ T cells that might explain their different homing preferences, we compared the expression of 266 surface markers using a flow cytometry-based screen developed by BioLegend (Supplementary Fig. 3b). Our analysis revealed 28 surface antigens that were differentially expressed between WT and TLR7[Tg] peripheral CD8^+^ T cells (Supplementary Fig. 3c; Fig. 3c). Remarkably, twelve of the total surface markers up- or down-regulated on peripheral TLR7[Tg] CD8^+^ T cells relative to WT controls are functionally required for either T cell adhesion to endothelium or retention in tissue (Fig. 3d). We next found that the changes in expression of nine of these twelve adhesion molecules were significantly upregulated on brain-infiltrating CD8^+^ T cells in TLR7[Tg] lupus-prone mice; namely CD49d, CD11a, CD31, CD54, CD49f, CD29, CD48, and CD43aag (Fig. 3e,f), as well as CD44 (Fig. 2a,b) and CD103 (Fig. 2c,d).

**Figure 4:**
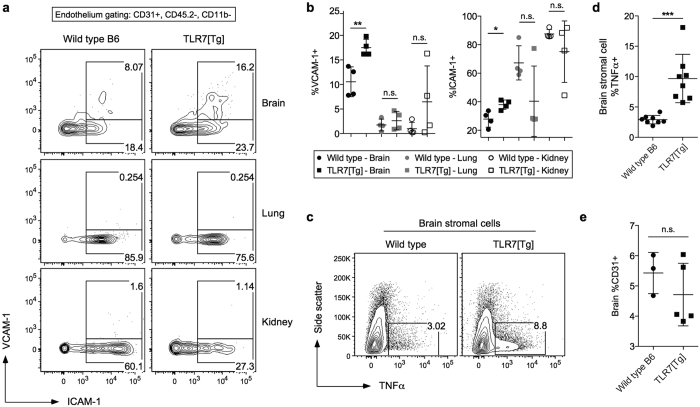
Activated brain endothelium in lupus-prone mice expresses cell adhesion molecules that support CD8^+^ T cell extravasation. (**a,b**) Percentage of CD31^+^ endothelium that express the adhesion molecules ICAM-1 and VCAM-1 in the brain, lung, and kidney of vessel labeled or trans-cardially perfused TLR7[Tg] and WT mice. Data are representative of three separate experiments. Gated on live CD45^**−**^CD11b^**−**^ cells. (**c,d**) Expression of TNFα by CD45^−^ stromal cells stimulated with phorbol 12-myristate 13-acetate (PMA) and ionomycin for five hours following isolation from trans-cardially perfused TLR7[Tg] and WT mice. Data in (**c**) are representative of two separate experiments. Data in (**d**) are pooled from both experiments. (**e**) Percentage of CD31^+^ endothelial cells from the brains of trans-cardially perfused TLR7[Tg] and WT mice. Based on gating in (**a,b**). Data are representative of three separate experiments. Gated on live CD45^**−**^CD11b^**−**^ cells. Error bars indicate mean + s.d. *P ≤ 0.05, **P ≤ 0.01, and ***P ≤ 0.001 (Student’s t test).

We next characterized the brain endothelium from WT and TLR7[Tg] mice for the expression of vascular adhesion molecules complementary to those observed on circulating CD8^+^ T cells (Fig. 3e,f). VLA-4 (CD49d) associates with either integrin β7 or CD29 (integrin β1) to bind VCAM-1 on tissue endothelium prior to extravasation^27^. LFA-1, a pairing of CD11a and CD18 proteins, as well as CD43aag, have both been shown to bind ICAM-1 and to be important for migration through activated endothelium^28^,^29^,^30^. We found that only the endothelium isolated from brain tissue, but not from lungs or kidneys had significant upregulation of both VCAM-1 and ICAM-1 (Fig. 4a,b). TNFα produced by brain stromal cells has been shown to drive expression of VCAM-1 and ICAM-1 on activated brain endothelia^31^. In accordance with these data we found that more stromal cells in the brains of TLR7[Tg] mice as compared to WT controls produced TNFα (Fig. 4c,d).

CD31 also plays a critical role in regulating T cell extravasation. In several models of autoimmunity, including experimental autoimmune encephalomyelitis^32^ and collagen-induced arthritis^32^,^33^,^34^, homophilic interaction of CD31 between lymphocytes and vascular endothelium of the CNS limits lymphocyte entry into the tissue. We did not find any change in CD31 expression between WT and TLR7[Tg] brain endothelium (Fig. 4e), but we did identify a marked decrease in the amount of CD31 on peripheral TLR7[Tg] CD8^+^ T cells compared to WT controls (Fig. 3e,f). Taken together, these data indicate that peripheral CD8^+^ T cells and the brain endothelium of lupus-prone mice are activated in a complementary manner, and support the notion of an inflammatory brain environment promoting leukocyte accumulation, adhesion, and migration from capillaries into the parenchyma.

### Brain-resident CD8^+^ T lymphocytes in lupus-prone mice are not pathogenic

**Figure 5:**
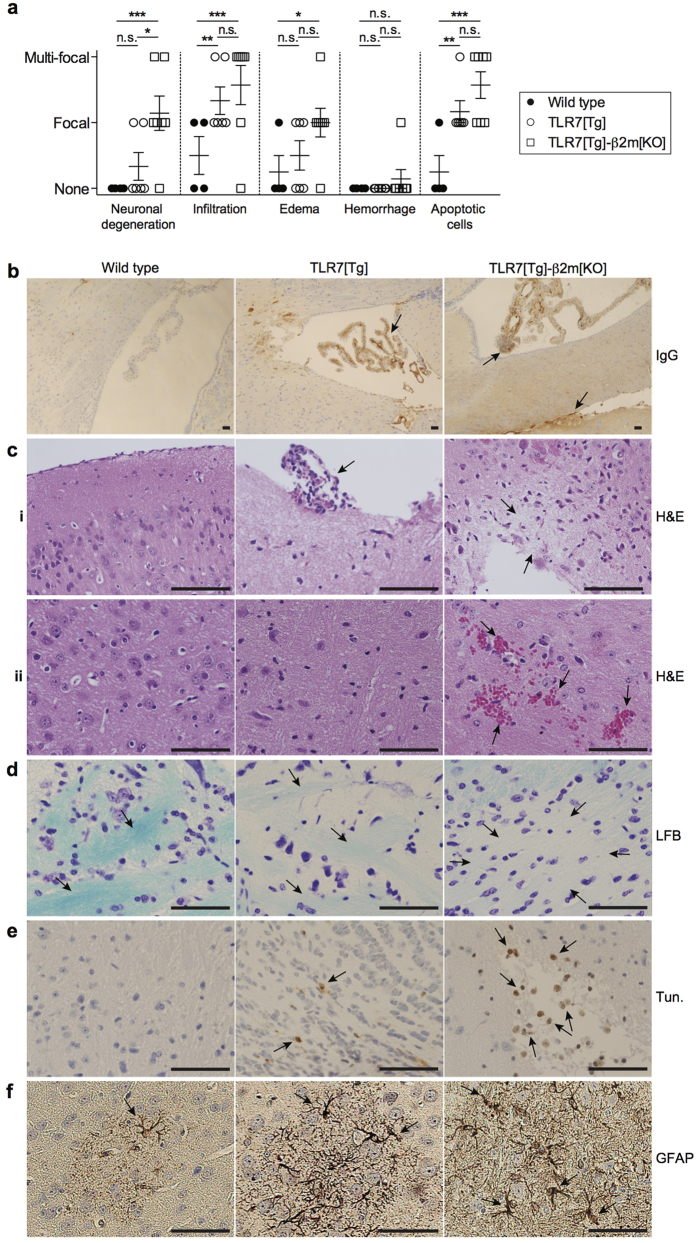
β2m deficiency accelerates CNS damage during murine lupus. Histopathological analysis of tissue sections from the brain of 4% formaldehyde perfused WT (n = 4), TLR7[Tg] (n = 6), and TLR7[Tg]-β2 m[KO] (n = 7). (**a**) Pathological phenotyping of indicated mice based on presence or absence of focal (single) or multi-focal (multiple) instances of neuronal degeneration, mononuclear cell infiltration, edema, focal hemorrhage, and apoptotic cells. (**b**) Brain endothelium barrier integrity measured by positive staining with anti-IgG antibody of brain tissue sections from indicated mice Scale bars, 50 μm. (**c**–**f**) Histopathological analysis of indicated mice by hemotoxin and eosin (H&E), Luxol Fast Blue (LFB), TUNEL (Tun.), and Glial Fibrillary Acidic Protein (GFAP). H&E shows architectural destruction, edema, and mononuclear cell infiltration (**c**, panel i), and microhemorrhages (**c**, panel ii). Loss of blue stain in LFB shows neuronal demyelination (**d**). TUNEL staining shows areas of apoptotic cell death (**e**). GFAP positive staining shows increased activation of astrocytes (**f**). Scale bars, 50 μm. Error bars indicate mean + s.d. *P ≤ 0.05, **P ≤ 0.01, and ***P ≤ 0.001 (one-way ANOVA, Tukey method).

To elucidate the role of the tissue-resident CD8^+^ T cells in lupus-prone mice, we bred TLR7[Tg] animals to germ-line deficient beta-2-microglobulin (β2m[KO]) animals. These mice lack the MHC class I adaptor molecule, β2m, which is required for normal development of CD8^+^ T lymphocytes and natural killer (NK) cells^35^. We confirmed that TLR7[Tg]-β2m[KO] mice were largely devoid of CD8^+^ T cells, both in the brain (Supplementary Fig. 4a) and in the spleen (Supplementary Fig. 4b). Next, we compared the total brain pathology in WT, TLR7[Tg], and TLR7[Tg]-β2m[KO] mice, which included analysis of barrier permeability to IgG, hematoxylin and eosin staining (H&E), Luxol Fast Blue (LFB), and TUNEL stains. Compared to WT controls, TLR7[Tg] brains showed some focal mononuclear cell infiltration and mild tissue destruction, but the β2m deficiency in TLR7[Tg] triggered instances of pathology that were not apparent in CD8-sufficient TLR7[Tg] mice, including multi-focal neuronal degeneration and edema, and hemorrhaging (Fig. 5a). TLR7[Tg] mice showed increased barrier permeability compared to WT brains, mostly around the third and fourth ventricles, as measured by immunohistochemical staining for total IgG. The IgG staining was even more pronounced in TLR7[Tg]-β2m[KO] brains (Fig. 5b). Relative to WT controls, TLR7[Tg] mice demonstrated instances of small architectural changes to the cortex and hippocampus, and meningeal lymphocyte infiltration indicated by H&E analysis, but no hemorrhaging. In contrast, TLR7[Tg]-β2m[KO] samples showed increased architectural changes including the emergence of mild edema highlighted by light colored fluid-filled lesions and multi-focal neuronal degeneration (Fig. 5ci), as well as the emergence of isolated microhemorrhaging (Fig. 5cii). Additionally, focal neuronal demyelination measured by LFB (Fig. 5d), and focal cell death measured by a TUNEL stain (Fig. 5e) each were apparent in TLR7[Tg] mice compared to WT controls, and further aggravated in TLR7[Tg]-β2m[KO] samples. Consistent with our data showing stromal cell activation (Fig. 4) in TLR7[Tg] mice we found the brains of these animals compared to WT controls also have small increases in glial fibrillary acidic protein (GFAP), an astrocyte specific protein which undergoes increased production in response to brain trauma (Fig. 5f).

**Figure 6:**
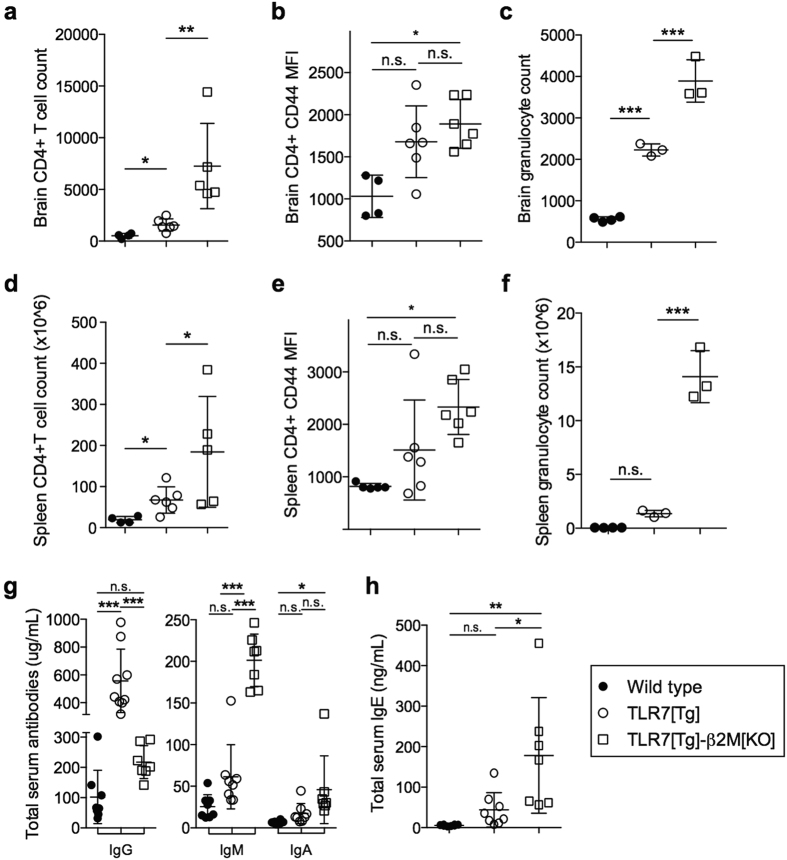
Increased serum IgE accompanies brain-infiltrating CD4^+^ T lymphocytes and granulocytes in lupus-prone mice that are also β2m-deficient. (**a,b**) Quantification of flow cytometry showing absolute number of CD4^+^ T cells in the brain of perfused WT, TLR7[Tg], and TLR7[Tg]-β2 m[KO] mice (**a**) and mean fluorescence intensity of the activation marker CD44 on brain CD4^+^ T cells from indicated mice (**b**). Data pooled from two experiments. Cells gated on live singlet CD45^+^. (**c**) Absolute number of granulocytes (CD45^+^Gr1^**hi**^CD11b^**hi**^) in the brain of WT, TLR7[Tg], and TLR7[Tg]-β2 m[KO] mice as determined by flow cytometry. Data are representative of three experiments. (**d,e**) Quantification of flow cytometry showing absolute number of CD4^+^ T cells in the spleen of WT, TLR7[Tg], and TLR7[Tg]-β2 m[KO] mice (**d**) and mean fluorescence intensity of the activation marker CD44 on spleen CD4^+^ T cells from indicated mice (**e**). Data are pooled from two experiments. Cells gated on live singlet CD45^+^. (**f**) Absolute number of granulocytes (CD45^+^Gr1^**hi**^CD11b^**hi**^) in the brain of WT, TLR7[Tg], and TLR7[Tg]-β2 m[KO] mice as determined by flow cytometry. Data are representative of three experiments. (**g,h**) Representative graphs of indicated serum antibody levels of WT, TLR7[Tg], and TLR7[Tg]-β2 m[KO] mice. Error bars indicate mean + s.d. *P ≤ 0.05, **P ≤ 0.01, and ***P ≤ 0.001 (one-way ANOVA, Tukey method).

The aggravated CNS pathology observed in TLR7[Tg]-β2m[KO] correlated with changes in immune cell populations detected in brain tissue. The CD45^+^ subset extracted from the brains of TLR7[Tg]-β2m[KO] contained a significantly higher number of CD4^+^ cells with increased expression of CD44, as well as more granulocytes compared to samples from TLR7[Tg] and WT control mice (Fig. 6a–c). These data implicate both CD4^+^ T cells and granulocytes as potential factors contributing to aggravated brain tissue pathology in lupus-prone mice in the absence of CD8^+^ T cells. As we have shown previously, TLR7[Tg] mice also have a substantial systemic inflammatory disease^21^, and so we decided to investigate whether peripheral CD4^+^ T cells or granulocytes were altered in TLR7[Tg]-β2m[KO] mice. We found substantial increases in total splenic CD4^+^ T cell numbers (Fig. 6d) and expression of CD44 (Fig. 6e), as well as an expansion of the splenic granulocyte population in TLR7[Tg]-β2m[KO] mice compared with TLR7[Tg] and WT animals (Fig. 6f), suggesting that CD8^+^ T cells could play a role in regulating peripheral immune activity in addition to brain pathology.

A previous report investigating the importance of MHC I proteins to disease in another lupus model, BXSB. Yaa, found global worsening of peripheral disease^36^. Consistent with these results, we found that the mortality of TLR7[Tg]-β2m[KO] mice was accelerated compared to TLR7[Tg] mice (Supplementary Fig. 4c), which correlated with the increase in brain pathology (Fig. 5), elevated hematopoietic infiltrates, and tissue damage in both the liver and kidney (Supplementary Fig. 4d,e). We also found more severe glomerulonephritis (Supplementary Fig. 4e), as well as increases in splenic follicle destruction (Supplementary Fig. 4f) and gross spleen weight (Supplementary Fig. 4g). In assaying for an effect of regulatory T cells in this model we found only marginal changes in the sizes of the CD8^+^Foxp3^+^ and CD4^+^Foxp3^+^ T cell pool in TLR7[Tg] spleens (Supplementary Fig. 4h). Surprisingly, we saw a significant increase in the total number of CD4^+^ regulatory T cells in the brain of TLR7[Tg]-β2m[KO] mice (Supplementary Fig. 4i) though the functional capacity of these cells remains untested. We also found that peripheral CD8^+^ T cells from TLR7[Tg] mice have elevated expression levels of the anti-inflammatory cytokine IL-10 relative to wild type controls (Supplementary Fig. 4j). Collectively these data suggest that neither brain-resident nor peripheral CD8^+^ T cells are pathogenic in this mouse model of lupus, rather genetic elimination of these populations by β2m deficiency is associated with exacerbation and not amelioration of pathology in the brain, kidney, liver, and spleen. Anti-inflammatory cytokine production by peripheral CD8^+^ T cells in these mice indicates a potential mean of suppressive capacity.

As both IgG and IgE autoantibodies have been implicated in lupus pathology^37^,^38^ we tested whether lupus-prone mice lacking CD8^+^ T cells had altered serum antibody levels of various isotypes. We found striking changes including a large decrease in total IgG, accompanied by increases in other antibody classes including IgM, IgA, and particularly IgE (Fig. 6g,h), indicating that skewed isotype switching in the absence of CD8^+^ T cells correlates with aggravated pathology.

## Discussion

Neuropsychiatric lupus is a debilitating but understudied aspect of SLE. Using two mouse models of spontaneous, progressive SLE, our study provides novel insight into the details of cellular immunity involved in CNS pathology during systemic inflammatory disease. Our work demonstrates that a population of CD8^+^ T lymphocytes uniquely accumulates in the CNS of lupus-prone mice and in no other organ examined, suggesting this is a specific and directed process.

The TLR7[Tg] and R2-Yaa models used in the present study each have a disease dependent on a Tlr7 gene dosage increase^21^. While the TLR7[Tg] animals do have more copies of Tlr7 relative to the R2-Yaa, we find similar infiltrating lymphocyte numbers and phenotype present in the brains of both models. Additionally, autoimmunity in TLR7[Tg] animals is dependent on CD40-CD40L signaling as Tlr7 overexpressing animals deficient in CD40L are completely protected from systemic disease^39^. Taken together, these data suggest that some lymphocyte-intrinsic effect is likely responsible for the neuropathology we see in TLR7[Tg] and TLR7[Tg]-β2m[KO] mice. Despite increased neuropathology in the absence of β2m there is no defect in the gross motor function or any outward signs of brain disease in these mice or β2m control littermates. We elected not to undertake behavioral phenotyping due to concerns that the rapid progression and severity of disease in TLR7[Tg]-β2m[KO] mice would make it difficult to interpret findings from any such assays. Despite this, our results are consistent with inflammation-induced damage of the brain barrier and tissue in lupus mouse models.

We provide evidence of non-pathogenic tissue resident-memory CD8^+^ T cells entering the brain through inflamed endothelia, and we confirm that these are truly tissue-resident and not vascular endothelial bound cells. Other groups have reported the presence of perivascular lymphocytes correlating with microvascular damage in the brain of human lupus patients^40^, as well as CD3^+^ lymphocytes in the choroid plexus and ventricles of MRL/lpr lupus-prone mice^18^,^41^, a model that has been used to study neuropsychiatric SLE^4^. These data suggest that the inflammatory state arising during both mouse and human lupus could induce conditions favorable to CNS lymphocyte entry. In line with this idea, we show an increase in total lymphocyte numbers in the CNS of two different mouse models of lupus, and we further demonstrate preferential and unique entry of a specific subpopulation of CD8^+^ T cells into the brains of these mice. TLR7[Tg] and R2-Yaa animals exhibit splenomegaly and peripheral lymphocyte expansion of B cells and CD4^+^ T cells, but no significant peripheral CD8^+^ T cell expansion^21^. We demonstrate that the age-dependent increase of CD8^+^ T cells in the brain could be due to either preferential retention or accumulation of lymphocytes, but not from changes in proliferation or cell death. While the peripheral CD8^+^ T lymphocytes in these mice demonstrate specific activation and tissue-homing surface expression characteristics, the brain-infiltrating population is enriched for the cells most representative of this phenotype. These data support the notion that there is selective recruitment and entry of a specific population of peripheral CD8^+^ T cells into the brain of lupus-prone mice that cannot be explained simply by overwhelming peripheral expansion and widespread inflammation, which would cause lymphocytes to spill into every organ.

Several mechanisms have been shown to contribute to tissue entry by lymphocytes that could explain the observed phenotype in our lupus-prone mice. For example, the importance of homophilic binding between CD31 on lymphocytes and endothelial cells in autoimmunity has been highlighted in experimental autoimmune encephalomyelitis and collagen-induced arthritis mouse models, in which genetic deletion of CD31 results in worsening of disease^32^,^33^,^34^. It is thought that CD31 serves as a circulation signal on T cells, restricting their entry into tissue. Consistent with this notion, we find substantial downregulation of CD31 on peripheral CD8^+^ T cells in lupus-prone mice and enrichment of this population within the brain parenchyma.

ICAM-1 and VCAM-1 are upregulated on activated tissue endothelium and known to be involved in controlling T cell entry into various organs through changes in their expression and ligation to their respective integrin receptors, LFA-1 (CD11/CD18a) and VLA-4 (CD49d/CD29)^42^. Both ICAM-1 and VCAM-1 have been specifically implicated in the control of lymphocyte entry into the brain following virally-induced encephalitis^43^ and in mouse and human studies of MS^44^. Other studies have found that ICAM-1 and VCAM-1 were upregulated in the ventricle region of the brains of MRL/lpr mice^45^, and that anti-ICAM-1 had a therapeutic effect, able to ameliorate abnormal neurological function in MRL/lpr mice^46^. In this study we show elevated brain endothelial expression of ICAM-1 and VCAM-1 and demonstrate that their expression is greater in the CNS than in other tissues in TLR7[Tg] mice. This finding corresponds with an enhanced ability of lupus brain stromal cells to make TNFα, which is recognized as the primary inflammatory cytokine responsible for inducing endothelial activation and expression of adhesion molecules^47^,^48^. The greatly elevated expression of the integrins LFA-1 and VLA-4 on CD8^+^ T cells in the periphery, and the concentration of these cells in the brain of our lupus-prone mice suggest that despite widespread systemic inflammation, there exists specificity and complementarity in the way the tissues and peripheral lymphoid compartment are activated.

Resident-memory T cells have been described both in regulation of immune responses within tissues following viral infection and also in settings of systemic inflammation and autoimmunity^20^. The CD8^+^ Trm cells defined in these different environments have some degree of phenotypic heterogeneity, and our brain-infiltrating T cells most closely resemble the CD69^hi^CD103^**−**^ Trm cells defined previously^25^,^49^,^50^,^51^. These cells were reported primarily to boost immune responses to pathogen in GFAP-specific CD8^+^ Trm cells found in the brain, and are associated with destruction of gray matter in a mouse model of MS^52^. In contrast to these findings, a study of freshly isolated cells from human brains has shown that CD8^+^ T cells from the corpus callosum lack cytolytic function and are proposed to have an immunoregulatory role^53^. Our data support the idea that CD8^+^ Trm cells can serve non-pathogenic roles in the tissues during autoimmune disease.

The study of regulatory CD8^+^ T cell has received more attention recently, though compared to CD4^+^ regulatory T cells, the CD8^+^ variety is less well understood. There is some evidence to suggest that CD8^+^ regulatory T cells play a role in controlling lupus^54^, and that these cells are defined by the marker CD122, the IL-2Rβ chain, among others^55^. One study citing a protective role for CD8^+^ T cells in a mouse model of lupus genetically ablated several distinct MHC class I family proteins including β2m, leading to a significant increase in disease in BXSB. Yaa lupus-prone mice^36^. This group also implicated the CD8^+^CD122^+^ population, suggesting it plays a key role in restricting expansion of CD4^+^ICOS^+^ T cells, antibody production, and development of B cell lymphomas. In addition, they suggest that NK cells might also contribute to this axis of protection as β2m also regulates the development of this lineage. Contrary to this idea, we recently found that genetic ablation of the IL-15 gene, which is required for normal NK cell development and function, ameliorates disease in TLR7[Tg] mice and suggests an exacerbating effect of cells of the NK cell lineage^56^,^57^. In the present study, we demonstrate that unlike TLR7[Tg] NK cells, the CD8^+^ T cells in these mice are not deleterious and may contribute to reducing systemic pathology. However, the brain-resident population does not match the phenotype (CD122^+^) of previously described regulatory CD8^+^ T cells, and they do not express Foxp3. Instead, the population we identify is similar to CD8^+^ Trm cells (CD122^**−**^CD103^**−**^) shown to regulate tissue immunity in the skin following bacterial infection, though the particular mechanism of action of these cells remains unclear^26^.

A potential mechanism by which tissue resident CD8^+^ T cells might regulate the immune response to self is by restricting the activity of components of the Th2-IgE axis. A Th2-type CD4^+^ T cell provides two signals to B cells to induce IgE class switching, namely CD40L costimulation and IL-4^58^. Th2 signals and IgE were recently shown to exacerbate lupus in humans^37^. In lupus-prone mice, elimination of IgE delays disease progression^38^, while genetic ablation of CD40L is completely protective^39^. We propose that in the absence of brain-resident CD8^+^ T cells, unrestricted CD4^+^ T cell activity and IgE production could be responsible for disease aggravation in lupus-prone mice. Interestingly, the high affinity IgE receptor, FcεRI, was recently shown to be expressed on neurons^59^, which indicates that any IgE entering the brain has the potential to induce a local inflammatory response. While it is clear that CD8^+^ T cells are required to regulate the antibody response in TLR7[Tg] lupus-prone mice, more studies will need to be performed to fully elucidate any regulatory mechanism that may be imparted by these brain-resident CD8^+^ T cells and determine if they are similar in function to memory cells previously described in models of viral or bacterial infection.

## Methods

### Mice and adoptive transfers

All animals used throughout this study, including TLR7.1[Tg], and FcγRIIB[KO]-Yaa^21^, and β2m[KO] (Taconic) mice, are on a C567BL/6J (WT) background. TLR7[Tg] and WT mice were also maintained on a CD45.1 congenic background. Mice were used between 8–14 weeks of age and results are representative of both male and female animals unless otherwise indicated. For adoptive transfers, splenic CD8^+^ T cells from donor TLR7[Tg] or WT control mice on the CD45.1 congenic background were purified by magnetic bead positive selection according to the manufacturer (RoboSep, Stem Cell). 5 × 10^6^ CD45.1^+^CD8^+^ T cells were injected IV, once per mouse. Organs of recipient mice were harvested by day six following injection following perfusion and prepared for flow cytometry as described below. All animals were housed and studied in accordance with the approved NIH Animal Study Protocol, and all experimental protocols were approved by and performed according to NIH ACUC guidelines. All efforts were made to minimize animal suffering and to reduce the number of animals used.

### Vascular labeling and perfusion

Discrimination of cells in the vasculature versus those in the tissue was performed as described^11^,^23^. 5 μg of either FITC- or BV421-CD45.2 (1D4, BioLegend) diluted in PBS was injected IV once per mouse. Antibodies were allowed to circulate for a maximum of three minutes and then mice were euthanized and organs extracted. Where indicated, trans-cardial perfusion with 30 ml ice-cold PBS was performed prior to organ extraction.

### Lymphocyte and endothelial cell extraction

Cell extraction from tissues was modified according to the described protocol^60^. For lymphocyte extraction, organs were digested for 30–60 minutes at 37 °C, and shaken at 240 rpm in HBSS containing 1 mg/ml Collagenase Type I (Life Technologies). Digested slurry was filtered and washed through a 70 μM mesh filter and resuspended in Percoll (GE Healthcare) diluted to 90% using HBSS. Layers of 60% and 37% Percoll were then sequentially overlayed on the 90% cell-Percoll mix. Cell separation was accomplished by centrifugation at 4 °C, 500 × g for 18 minutes with the brakes off. Cells were washed and resuspended in staining buffer containing 5% FBS in PBS in preparation for flow cytometry. Neural cells extraction was performed using previously described methods^60^. Briefly, cells were digested using 0.8 mg/ml Dispase 2 (Sigma), 0.2 mg/ml collagenase P (Sigma), and 0.1 mg/ml DNase I (Sigma). Density separation of cells was done in one step using only 40% Percoll, and subsequent pelleted cells were filtered and washed through a 100 μM filter before preparation for flow cytometry.

### Flow cytometric analysis

Single-cell solutions were prepared from organs as indicated and resuspended in staining buffer containing 5% FBS in PBS. Splenic and tissue-resident lymphocytes were identified with mAbs against the following antigens, all from BioLegend unless otherwise noted: CD8α (MAR-1), CD4 (RM4-5), CD19 (6D5), B220 (RA3-6B2), NK1.1 (eBiosciences, PK136), Gr1 (eBio, RB6-8C5), CD11b (M1/70), CD45.2 (1D4), CD45.1 (A20), CD44 (BDIM7) CD62L (MEL-14), CD45RB (eBio, C363.16a), CD69 (BD Biosciences, H1.2F3), PD-1 (29F.1A12), CD103 (2E7), Ly-6C (HK1.4), CD122 (5H4), CD49d/VLA-4 (R1-2), CD11a (M17/4), CD43aag (1B11), CD29 (HMβ1-1), CD48 (HM48-1), CD49f/VLA-6 (GoH3), CD31 (390), ICAM-1 (YN1/1.74), VCAM-1 (429), TCRβ, (eBio, H57-597). Live and dead cells were separated using Zombie Aqua^TM^ Fixable Viability Kit (BioLegend). Data were acquired on an LSR II SORP (BD) equipped with a Violet (406 nm, 100 mW), Blue (adjustable 488 nm, 80 mW; maximum output 100 mW), Green (532 nm, 150 mW) and Red (642 nm, 40 mW) lasers, then analyzed with FlowJo (TreeStar Technologies).

### Intracellular cytokine and transcription factor analysis

For intracellular detection of *in vitro* cytokine and transcription factor production, cells were plated at a density of 2 × 10^6^ ml in RPMI1640 (Gibco) containing 10% FBS, L-glutamine, and 2-mercaptoethanol. Cells were stimulated for 4-6 hours using either 1 μg/ml each, plate-bound anti-CD3ε f(ab′)2 (2C11, BioXCell), and anti-CD28 (37.51, BioXCell) in the presence of GolgiStop (3 mM monensin, BD), or Cell Stimulation Cocktail plus protein transport inhibitor (50 ng/ml phorbol 12-myristate 13-acetate (PMA), 1.34 mM ionomycin, 5.3 mM brefeldin A, 1 mM monensin) according to manufacturer guidelines (eBio). After surface staining, cells were fixed and permeabilized according to manufacturer guidelines (BD). Subsequent intracellular staining was performed with anti-TNFα (BD, MP6-XT22). Foxp3 (Fjk-16s, eBio) was detected in lymphocytes *ex vivo* following fixation and permeabilization according to manufacturer guidelines (eBio).

### Histology and pathological analysis

Organs were fixed in 10% buffered formalin, and embedded in paraffin following trans-cardial perfusion with 4% formaldehyde (Sigma). The tissue sections were stained either by hematoxylin and eosin (H&E) or by immunostaining according to standard protocols, and examined by light microscopy with magnifications of 2X, 5X, 10X, 20X, and 40X. The microscopic images were then evaluated by a pathologist. Following hematoxylin staining of tissues, immunostaining for CD3 (MCA1477, AbD Serotec) was performed by the avidin-biotin peroxidase complex (ABC) method using the Vectastain Elite ABC kit (Vector Labs) according to manufacturer instructions as described previously. Staining for GFAP (Z0334, DAKO), as well as LFB (56621, Sigma) were used following the standard staining procedure. For apoptosis detection a standard TUNEL (Terminal deoxynucleotidyl transferase dUTP nick end labeling) assay was performed. Briefly, following deparaffinization and rehydration of slides, sections were treated with Proteinase K and H_2_O_2_/PBS to block the endogenous peroxidase activity. Slides were then incubated with TdT (M0315L, New England Biolabs) and the reaction was detected with antidigoxigenin-peroxidase (11207733910, Roche Diagnostics) and DAB. Slides were counterstained with hematoxylin and coverslipped for microscopy observation. Brain endothelial integrity was qualified by immunostaining with anti-IgG. The slides were stained using LeicaBiosystems Bond Autostainer with Bond™ Intense R Detection Kit, according to manufacturer instructions. Briefly, the slides were treated in citrate for 20 minutes for antigen retrieval and then in 2% normal horse serum for another 20 minutes. The slides were then incubated with biotinylated horse anti-mouse IgG antibody (Vector Labs, 1:100 in 1.5% serum) for 30 minutes and subjected to microscopic analyses. All samples were analyzed and scored blindly by two pathologists. Sandwich ELISA for total serum antibody concentration of IgG, IgM, IgA was performed using goat anti-mouse Ig H+L chain capture antibody, unlabeled mouse IgG, IgM, or IgA antibodies for standard curve, and corresponding goat anti-mouse AP detection system, all according to manufacturer (Southern Biotech) instructions. Specific IgE sandwich ELISA was performed using anti-mouse IgE capture antibody (BD Pharmigen, R35-72), and biotin-streptavidin detection system, and was otherwise identical to other antibody ELISA described above.

### Statistical analysis

Statistical significance of data was calculated with Prism 6.0 software (GraphPad). For comparisons between two normally distributed groups a two-tailed unpaired *t*-test with Welch’s correction was used. Non-parametric data was analyzed using Mann-Whitney *U* test. For comparison between more than two groups statistical analysis was performed using a one-way ANOVA with the Tukey method. Groups of biological replicates pooled across multiple experiments were compared using two-way ANOVA. Differences in mortality rates of mice were assessed by Cox-Mantel log rank analysis. Error bars indicate mean + s.d. *P ≤ 0.05, **P ≤ 0.01, and ***P ≤ 0.001 (Student’s t test).

## Additional Information

**How to cite this article**: Morawski, P. A. *et al*. Non-pathogenic tissue-resident CD8^+^ T cells uniquely accumulate in the brains of lupus-prone mice. *Sci. Rep.* **7**, 40838; doi: 10.1038/srep40838 (2017).

**Publisher's note:** Springer Nature remains neutral with regard to jurisdictional claims in published maps and institutional affiliations.

## Supplementary Material

Supplementary Information
