# Supplementary material for: Non-pathogenic tissue-resident CD8+ T cells uniquely accumulate in the brains of lupus-prone mice
Source: Sci Rep. 2017 Jan 18;7:40838. doi: 10.1038/srep40838 (PMC5241651; doi:10.1038/srep40838)
Supplement: Supplementary Information [file srep40838-s1.pdf]

Supplementary Information

Non-pathogenic tissue-resident $CD8^+$ T cells uniquely accumulate in the brains of lupus-prone mice

Peter A. Morawski[1], Chen-Feng Qi[1], and Silvia Bolland[1*]

[1]Laboratory of Immunogenetics, Division of Intramural Research, National Institute of Allergy and Infectious Diseases, NIH, Rockville, MD 20852, USA

[*]Correspondence: sbolland@niaid.nih.gov

Supplementary Figure 1 | T lymphocytes accumulate uniformly in the brain and spinal cord of TLR7[Tg] mice.

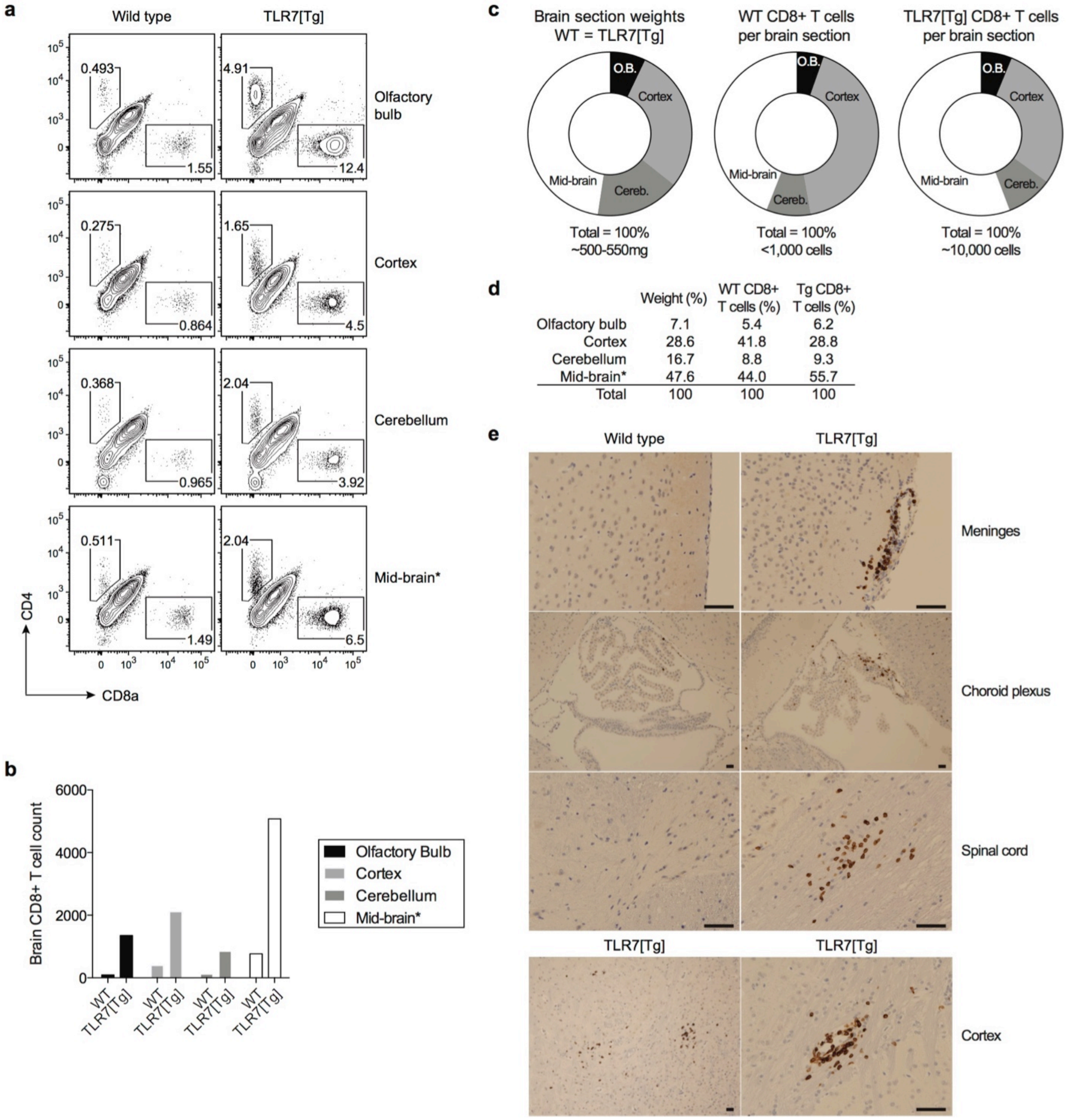

(**a**) Brains from trans-cardially perfused WT and TLR7[Tg] mice were dissected into four sections: olfactory bulbs, cortex, cerebellum, and mid-brain*. The mid-brain represents all remaining unrepresented deeper parts of the brain including the thalamus, hypothalamus, and

corpus callosum. Flow cytometry analysis of $CD8^+$ and $CD4^+$ T lymphocytes was performed on isolated mononuclear cells from indicated brain sections. Cells gated on live, singlet, $CD45^+$. Data shown include five pooled sections of brains for each WT and TLR7[Tg] mice. (**b**) Absolute $CD8^+$ T cell count for indicated brain sections enumerated based on flow cytometry analysis in (**a**) comparing WT and TLR7[Tg] mice. (**c, d**) Weights of brain sections from (**a**) represented as parts of whole (left chart), where 100% is equal to the average mouse brain weight (~500-550mg for both WT and TLR7[Tg] mice), and $CD8^+$ T cell counts as parts of whole per brain section (middle chart, WT; right chart, TLR7[Tg]) where 100% is equal to the total average enumerated $CD8^+$ T cell count per brain (<1,000 for WT and >10,000 for TLR7[Tg] mice). (**d**) Summary of data. (**e**) Immunohistochemical staining of brain tissue for hematoxylin and CD3 from 4% formaldehyde perfused TLR7[Tg] and WT mice confirms the presence of lymphocytes throughout the CNS including meninges, choroid plexus, spinal cord, and cortex. Scale bars, 50 μm. Data are of a representative experiment of five mouse brains dissected into indicated sections and pooled to provide a number of T cells sufficient for analysis.

Supplementary Figure 2 | Accumulation of CD8⁺ T lymphocytes in the brain of lupus-mice is not caused by changes in cell proliferation or cell death.

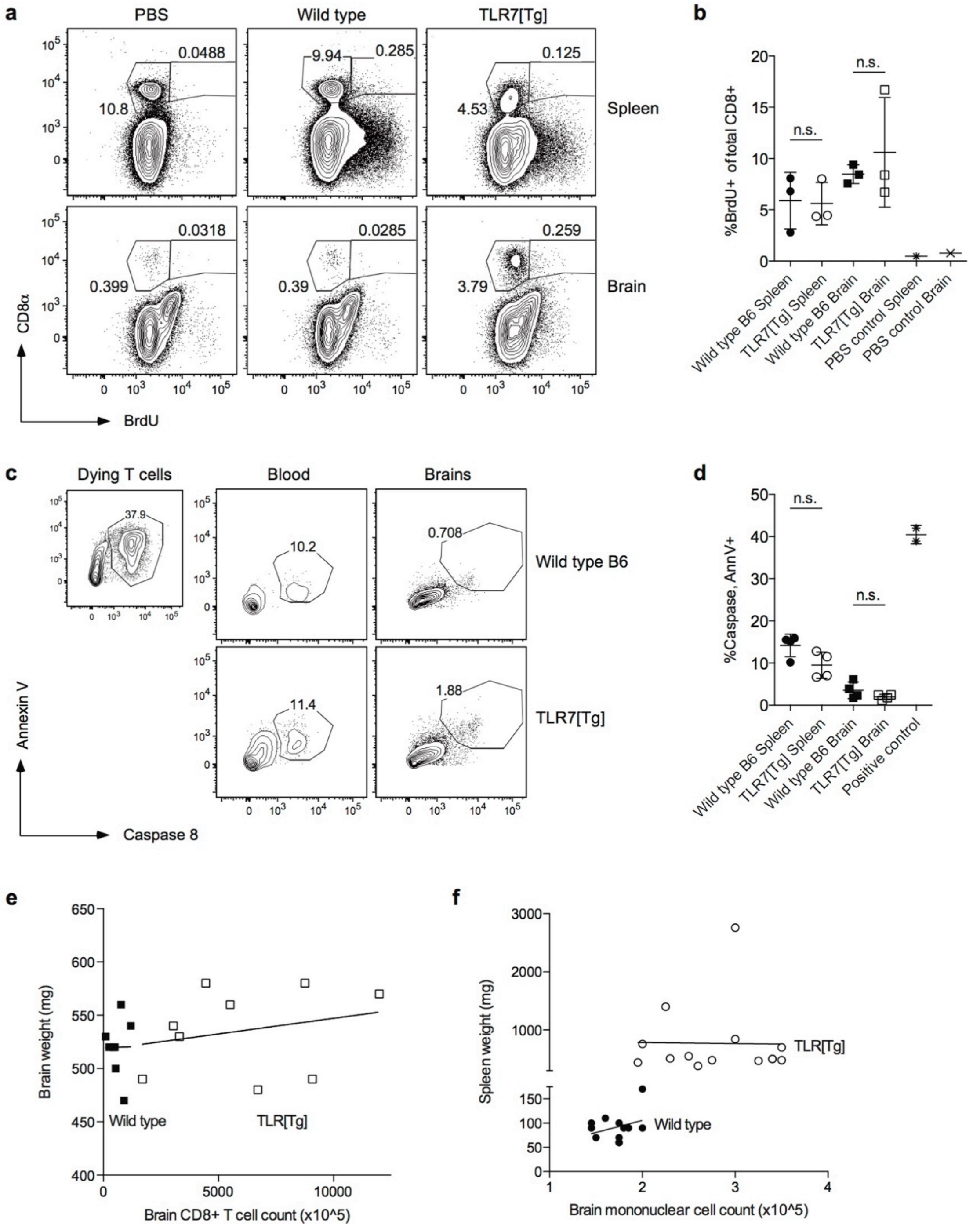

(**a, b**) WT and TLR7[Tg] mice were injected with BrdU every other day for a week. Organs were harvested after trans-cardial perfusion eight days following first injection. Flow cytometry

analysis of BrdU$^+$ CD8$^+$ T cells isolated from spleen and brain shown. Cells gated on live, singlet, CD45$^+$. Data shown is representative of two experiments. (**c, d**) Peripheral blood was collected, then WT and TLR7[Tg] mice were perfused. Isolated mononuclear cells from peripheral blood and brain were stained with caspase 8 and annexin V to assess cell death. Dying T cells indicate positive control of peripheral blood cultured in serum free medium for one hour. Cells are gated on singlet, CD45$^+$. Data shown is representative of two experiments. (**e, f**) Organs were removed from trans-cardially perfused WT and TLR7[Tg] mice and weighed prior to preparation of mononuclear cells for flow cytometry as described. Absolute numbers of CD8$^+$ T cells plotted against brain weight (**e**), and total brain mononuclear cells plotted against spleen weight (**f**) shown. Data are pooled from three experiments. Error bars indicate mean + s.d. $^*P \leq 0.05$, $^{**}P \leq 0.01$, and $^{***}P \leq 0.001$ (Student's t test).

Supplementary Figure 3 | Analysis of non-pathogenic peripheral lymphocytes from TLR7[Tg] lupus-prone mice

a

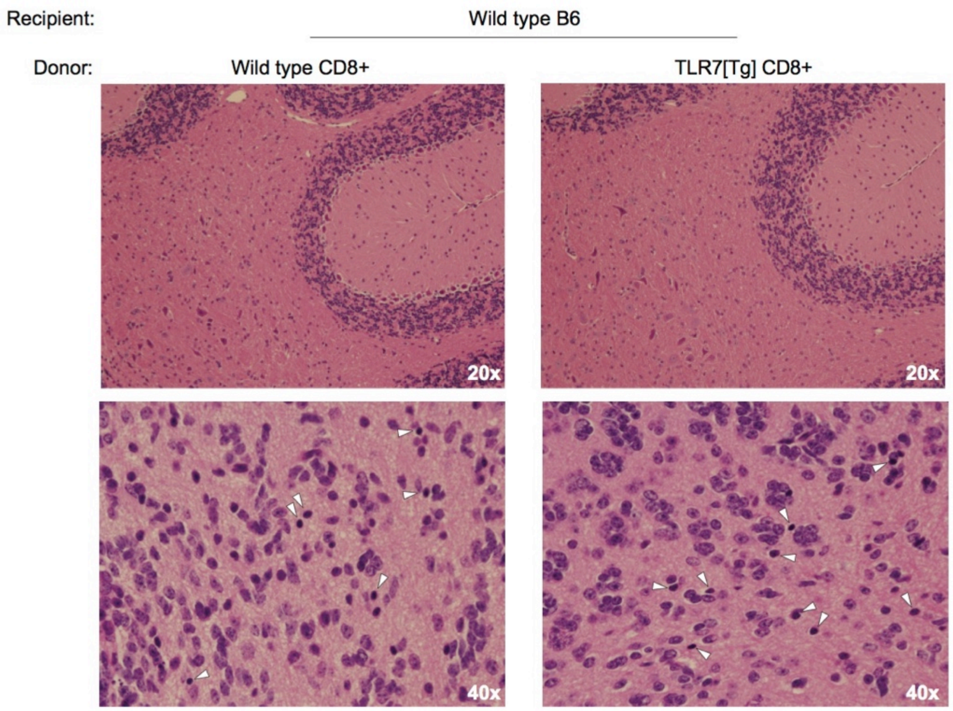

b

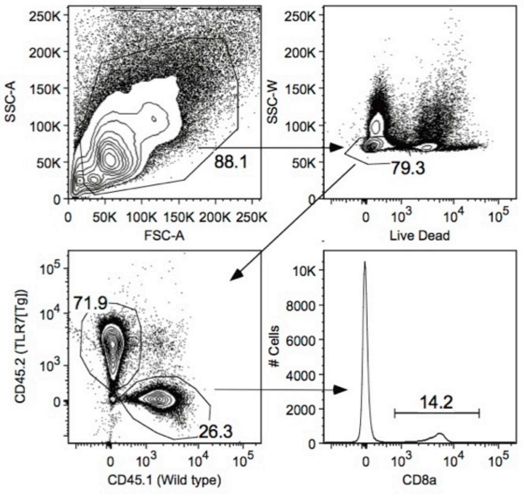

c

Antigen	Fold over WT	Function
Ly-6C	2.79	activation, cd8 memory
CD81	2.25	t cell growth
CD38	2.20	ADP-ribosyl cyclase
CD27	2.11	costimulation
CD43	2.11	adhesion/activation
CD29	1.73	adhesion
CD44	1.67	activation, adhesion
CD183	1.66	chemokine R
CD54	1.58	adhesion
CD278	1.55	costimulation
CD55	1.53	complment regulation
CD11a	1.51	adhesion
CD18	1.47	adhesion
CD86	1.47	activation, costimulation
CD48	1.41	adhesion
H-2	1.41	antigen presentaiton
CD122	1.39	inhibitory CD8s
CD49f	1.37	adhesion
CD49d	1.29	adhesion
CD5	1.26	inhibits B-T interaction
CD8a	-1.16	co-receptor
CD45RB	-1.17	activation
CD2	-1.27	adhesion/activation
CD3	-1.37	activation
TCR β	-1.52	antigen recognition
CD31	-1.53	adhesion/extravasation
CD197	-1.54	trafficking
Integrin β7	-1.68	epidermal/LN retention
CD127	-1.71	proliferation
CD199	-1.78	trafficking
CD103	-2.22	tissue resident memory

(**a**) Two successive intravenous adoptive transfers were performed on day 1 and day 8 of 3x10^6 purified CD8+ T cells from either wild type B6 or lupus-prone TLR7.1[Tg] animals into healthy B6 animals, n=5 per group. Donor cells were taken from congenic animals to allow for discrimination from host cells. On the 12th day following initial injection brains from all mice were prepared for H&E pathological analysis. White arrowheads indicate presence of lymphocytes. No significant pathological abnormalities were identified in either wild type or TLR7[Tg] animals. (**b,c**) Splenocytes from WT CD45.1 and TLR7[Tg] CD45.2 mice were mixed and prepared for BioLegend Screen as described. Data represent a single use of the screening kit according to manufacturer instructions. (**b**) Gating strategy for separating WT and TLR7[Tg] congenically labeled, live, CD8^{+} T cells prior to analysis of multiple-antigen PE screen. (**b**) Following subgating in (**c**), candidates from multi-antigen screen were pre-selected as described based on mean fluorescence intensity of each screened antigen in the PE channel. The ratio of TLR7[Tg] to WT expression of each PE-labeled antigen is shown as fold over WT. Conformational analysis was performed and relative expression between WT and TLR7[Tg] peripheral lymphocytes compared to TLR7[Tg] brain-resident lymphocytes is shown in Figure 3.

Supplementary Figure 4 | Disease in TLR7[Tg] lupus-prone mice is aggravated globally in the absence of MHC class I adaptor protein β2m.

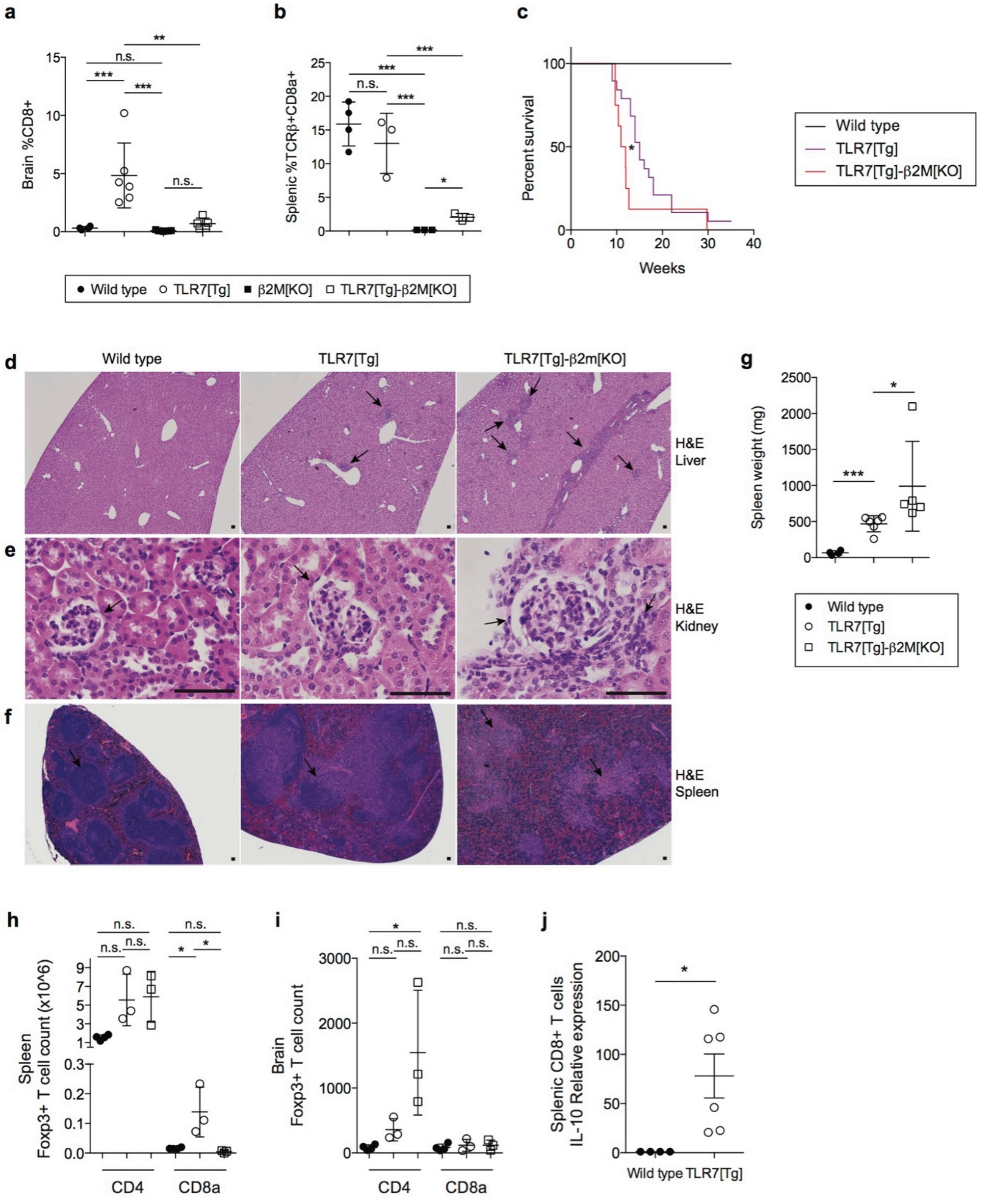

(**a, b**) Quantification of flow cytometry showing percentage of total hematopoietic cells expressing TCRβ and CD8α in the brain (**a**) and spleen (**b**) from trans-cardially perfused WT, TLR7[Tg], and TLR7[Tg]-β2m[KO] mice. Data pooled from two experiments. Cells gated on live singlet, $CD45^{+}$. (**c**) Survival curves for WT (n=8), TLR7[Tg] (n=12), and TLR7[Tg]-β2m[KO] (n=7). (**d-f**) Histopathological analysis of peripheral tissues from indicated mice perfused with 4% formaldehyde. Arrows indicate lymphocyte infiltrates of liver (**c**), inflamed and damaged glomeruli in the kidney (**d**), or destruction of splenic follicles with blasting germinal center cells (**e**) Scale bars, 50 μm. Analysis of brain tissue from these mice is performed in Figure 5. (**g**) Spleen weight of WT, TLR7[Tg], and TLR7[Tg]-β2m[KO] pooled from two separate experiments. (**h, i**) Absolute number of $CD4^{+}$ and $CD8^{+}$ T cells that are $Foxp3^{+}$ in the spleen and brain of WT, TLR7[Tg], and TLR7[Tg]-β2m[KO] animals, representative of two separate experiments. (**j**) IL-10 gene expression of *ex vivo*, splenic $CD8^{+}$ T cells from WT and TLR7[Tg] mice. IL-10 relative expression is calculated against 18S ribosome control using δ-δ Ct method. Data are pooled from two separate experiments. Differences in mortality rates of mice were assessed by Cox-Mantel log rank analysis. Error bars indicate mean + s.d. $^{*}P \leq 0.05$, $^{**}P \leq 0.01$, and $^{***}P \leq 0.001$ (one-way ANOVA, Tukey method). For comparisons between two normally distributed groups a two-tailed unpaired *t*-test was used.

Supplementary Methods

***In vivo* lymphocyte expansion.** Mice were injected with 1 mg Bromodeoxy-Uridine (BrdU) (Roche) IP in 200 µl PBS every other day for eight days. Animals were sacrificed following perfusion with 1% PBS. Indicated organs were harvested and single-cell solutions were prepared for flow cytometric analysis as described.

Lymphocyte apoptosis. Single-cell preparations were prepared for flow cytometry from indicated organs. Cells engaging programmed death pathway were identified using CaspGlow™ fluorescein active caspase-8 staining kit (casp8/annexin V, eBiosciences) according to the manufacturers instructions. As a positive control, cell death was induced by culturing lymphocytes in serum-free media at 37°C for 1 hour.

Lymphocyte surface antigen screen and pre-processing of data. The Mouse BioLegend Screen™ was performed according to manufacturer instructions (BioLegend). A single-cell suspension of mixed WT CD45.1 and TLR7[Tg] CD45.2 congenic spleens was prepared. Mixed lymphocytes were added to 96-well plates provided by manufacturer containing lyophilized PE-labeled antibodies against 266 surface antigens and appropriate isotype controls. Cells were further stained with a multi-color panel for analysis by flow cytometry. Mean fluorescence intensity (MFI) of all surface antigens (PE labeled) was compared on gated $CD8^+$ T lymphocytes, and candidates with significant differences were selected based on a minimum MFI of 50 and a fold change of TLR7[Tg] over WT of 1.1x. Grouping was then performed based on known

functions of selected antigens. Subsequent validation was performed (n=3+ mice) with BioLegend antibodies of the same clones.

Quantitative PCR analysis of mRNA transcripts. $CD45^{+}CD8^{+}CD4^{-}$ T cells were purified from mononuclear preparations from the spleen of wild type or TLR7[Tg] mice by fluorescence cytometry sorting. Total RNA was extracted using RNeasy reagent kit (Qiagen), and reverse transcription was performed using the iScript Select cDNA synthesis kit (BioRad). Specific mRNA transcripts were quantified using SYBR Green (BioRad) on a CFX Connect real-time thermal cycler (BioRad). Expression levels were calculated for each sample by first normalizing to 18S rRNA and then normalizing to unstimulated wild type $CD8^{+}$ T cells using the standard δ-δ CT method. IL-10 forward, 5'-GATTTTAATAAGCTCCAAGACCAAGGT-3', IL-10 reverse, 5'-CTTCTATGCAGTTGATGAAGATGTCA-3', 18S rRNA forward, 5'-TTCGAACGTCTGCCCTATCAA-3', 18S rRNA reverse, 5'-ACCCGTGGTCACCATGGTA-3'.
